# Modeling Nitrogen Losses in Conventional and Advanced Soil-Based Onsite Wastewater Treatment Systems under Current and Changing Climate Conditions

**DOI:** 10.1371/journal.pone.0158292

**Published:** 2016-06-29

**Authors:** Ivan Morales, Jennifer Cooper, José A. Amador, Thomas B. Boving

**Affiliations:** 1 Department of Civil and Environmental Engineering, University of Rhode Island, Kingston, Rhode Island, United States of America; 2 Laboratory of Soil Ecology and Microbiology, University of Rhode Island, Kingston, Rhode Island, United States of America; 3 Department of Geosciences, University of Rhode Island, Kingston, Rhode Island, United States of America; Purdue University, UNITED STATES

## Abstract

Most of the non-point source nitrogen (N) load in rural areas is attributed to onsite wastewater treatment systems (OWTS). Nitrogen compounds cause eutrophication, depleting the oxygen in marine ecosystems. OWTS rely on physical, chemical and biological soil processes to treat wastewater and these processes may be affected by climate change. We simulated the fate and transport of N in different types of OWTS drainfields, or soil treatment areas (STA) under current and changing climate scenarios, using 2D/3D HYDRUS software. Experimental data from a mesocosm-scale study, including soil moisture content, and total N, ammonium (NH_4_^+^) and nitrate (NO_3_^-^) concentrations, were used to calibrate the model. A water content-dependent function was used to compute the nitrification and denitrification rates. Three types of drainfields were simulated: (1) a pipe-and-stone (P&S), (2) advanced soil drainfields, pressurized shallow narrow drainfield (PSND) and (3) Geomat (GEO), a variation of SND. The model was calibrated with acceptable goodness-of-fit between the observed and measured values. Average root mean square error (RSME) ranged from 0.18 and 2.88 mg L^-1^ for NH_4_^+^ and 4.45 mg L^-1^ to 9.65 mg L^-1^ for NO_3_^-^ in all drainfield types. The calibrated model was used to estimate N fluxes for both conventional and advanced STAs under current and changing climate conditions, i.e. increased soil temperature and higher water table. The model computed N losses from nitrification and denitrification differed little from measured losses in all STAs. The modeled N losses occurred mostly as NO_3_^-^ in water outputs, accounting for more than 82% of N inputs in all drainfields. Losses as N_2_ were estimated to be 10.4% and 9.7% of total N input concentration for SND and Geo, respectively. The highest N_2_ losses, 17.6%, were estimated for P&S. Losses as N_2_ increased to 22%, 37% and 21% under changing climate conditions for Geo, PSND and P&S, respectively. These findings can provide practitioners with guidelines to estimate N removal efficiencies for traditional and advanced OWTS, and predict N loads and spatial distribution for identifying non-point sources. Our results show that N losses on OWTS can be modeled successfully using HYDRUS. Furthermore, the results suggest that climate change may increase the removal of N as N_2_ in the drainfield, with the magnitude of the effect depending on a drainfield type.

## Introduction

Decentralized wastewater treatment systems, such as onsite wastewater treatment systems (OWTS), are engineered technologies for wastewater management practices that protect public health and lower contamination risk. Onsite wastewater treatment systems integrate a septic tank, where solids removal takes place, and a soil treatment area (STA), or drainfield, where contaminants are attenuated and treated wastewater infiltrates to safely recharge groundwater. Passage of wastewater through the STA of conventional system attenuates the 5-day biochemical oxygen demand (BOD_5_), total suspended solids (TSS), pathogens and nutrients (i.e. N, P). Conventional systems are not designed for removal of nitrogen (N) [1,2] or emerging organic contaminants, such as personal care products and pharmaceuticals [3,4]. Furthermore, they are less effective in areas with a shallow water table, and in coastal areas. Advanced OWTS are used in areas that are at risk of water use impairments (i.e., pathogen and nutrient contamination) because of a shallow-placed infiltrative surface.

Most conventional STAs receive septic tank effluent (STE). These systems have a pipe-and-stone (P&S) configuration: a horizontal drain constructed from perforated pipes placed in an excavated trench backfilled with gravel or crushed stone. Advanced OWTS integrate engineered treatment units (i.e., sand filters, aeration units) that provide additional treatment. The advanced treated effluent (ATE) can then be pressure-dosed to an STA place closer to the surface than that of a conventional OWTS. Such a system is known as pressurized shallow narrow drainfield (PSND). In advanced and conventional OWTS, the STA is dosed with STE or ATE. These drainfields are usually installed 15–30 cm below the ground surface (bgs) for advanced OWTS and ~ 60 cm bgs for conventional systems [5]. The shallow depth of the STA of advanced OWTS increases the vertical separation distance, or thickness of the unsaturated zone, and enhances the potential for treatment before the effluent reaches the water table [6–8]. A thicker unsaturated zone also increases O_2_ diffusion and attenuation of contaminants [9–12]. Another advantage of PSND over conventional STAs is that pressurized systems disperse the effluent more uniformly over the treatment area, which avoids overloading (ponding) and promotes complete infiltration [13]. A shallow drainfield also enhances the transformation of nutrients by microorganisms and their uptake by plants because effluent distribution takes place closer to the soil surface, within the root zone where microbial activity is highest [7].

Onsite wastewater treatment systems are sources of surface and groundwater contamination. They are one of the top 10 probable sources of impairments of rivers, lakes, and the coastal shoreline by pathogens and nutrients in the U.S. [14]. Nitrogen from OWTS is of particular concern because its presence in high concentrations has a negative impact on surface and coastal water ecosystems. For instance, approximately 32% of U.S. rivers and streams are considered stressed or affected by N [15–17]. Excess N in coastal areas and some freshwater ecosystems can result in eutrophication, followed by decreased dissolved oxygen levels and habitat degradation [15–17]. Nitrogen in wastewater is present primarily as organic nitrogen, ammonium (NH_4_^+^), and nitrate (NO_3_^-^) [18]. The nitrogen speciation in OWTS effluent depends on the type of system. In conventional systems, the STE is typically composed of 10–30% organic nitrogen and 70–90% NH_4_^+^ [5,19]. In ATE the typical N speciation is 18% organic N, 26% NH_4_^+^ and 56% NO_3_^-^ [20].

As STE and ATE are dispersed to the drainfield, N species can be transformed or removed in the soil below the infiltrative surface. Nitrogen transformations in conventional and advanced STA have been studied to some extent [21]. Nitrification and denitrification are thought to be the main processes that contribute to N speciation in the drainfields [22]. Nitrification involves the oxidation of NH_4_^+^ by autotrophic bacteria to NO_3_^-^ under oxic conditions. Nitrate can be subsequently reduced under anoxic conditions by heterotrophic denitrifying bacteria to nitrogen gas (N_2_) or nitrous oxide (N_2_O), both of which result in net removal of N from wastewater.

The fate and transport of N in OWTS drainfield is a complex process controlled by temperature, moisture content, carbon availability, oxygen diffusion and other factors. A suite of computer-aided numerical models have been developed to simulate N dynamics in the STA. However, many of these only modeled the fate of NO_3_^-^ in groundwater and hydrodynamic processes (advection-dispersion) [21–23]. Others used HYDRUS 1D, 2D and 3D models to predict the fate and transport of N in OWTS [24–27]. HYDRUS is a commercially-available computer program used to simulate water flow, solute, heat and colloid and microbial transport in variably-saturated porous media [28–30]. For instance, Hassan [24] used HYDRUS 2D to simulate an onsite wastewater subsurface drip irrigation system (SDIS) dosed with advanced-treated wastewater in a sequential batch reactor (SBR). The wastewater was collected from a restaurant and contained oil and grease with high organic C content. Together with a grease trap and aeration unit, the SBR was used as a pre-treatment unit, where NH_4_^+^ was nitrified and entered the SDIS as NO_3_^-^. The model included NO_3_^-^ transport, plant uptake, and denitrification in order to estimate an N mass balance for the SDIS-SBR system. In addition, the soil water pressure head data was modeled. It was estimated that 48% of NO_3_^-^ was stored in the soil profile, 27% was taken up by plants, 22% removed by denitrification, and 0.4% NO_3_^-^ left with the drainage water.

Heatwole and McCray [25] used HYDRUS 1D to model the fate and transport of nitrogen species in a conventional STA. The model evaluated the concentration of NO_3_^-^ reaching groundwater using site-specific data. The authors relied on input transport parameters estimated from statistical distributions of first-order nitrification and denitrification rate coefficients, dispersivity, effluent loading rate and nitrogen effluent concentration. The model predicted that no NH_4_^+^ should be detected at or below 30-cm from the infiltrative surface. Also, NO_3_^-^ concentrations were predicted to be below maximum contaminant level (MCL = 10 mg NO_3_^-^−N/L) when a median value for denitrification rate (0.042 day^-1^) was applied.

HYDRUS 2D/3D was also used to fit experimental soil pressure head and N and chloride (Cl^-^) data collected from a conventional OWTS with a drainfield installed in a clay soil [26]. The model involved the application of an N transformation chain, that is non-equilibrium transport of N in sequential decay reactions (NH_4_^+^ → NO_3_^-^→ N_2_) with water content-dependent, first-order transformation rates for nitrification and denitrification. Unlike Heatwole and McCray [25], the model assumed that N decay occurs and aquifer recharge was considered. The authors computed N losses from the STA with the calibrated model. Based on a N mass balance, the model predicted that 52% of N was removed by denitrification. Furthermore, less than 5% of N loss occurred as plant uptake and change in N storage. The model [26] was then used by Radcliffe and Bradshaw [27] to evaluate OWTS hydraulic loading rates (HLR) and N transformations in 12 soil textural classes. As in the previous study [26], water flow and N and temperature dynamics were simulated in a 2-D drainfield trench for a two-year period. All HLRs values (range: 1.48 to 5.40 cm d^-1^) were found to be suitable for all soil types except for the sandy clay textural class, where the trench was overloaded (HLR = 1.48 cm d^-1^). The predictions for denitrification losses varied widely among soil types, from 1% in sand to 75% in sandy clay. Leaching losses of NO_3_^-^ were more significant than denitrification, ranging from 27% in sandy clay to 97% in sand. The variations in leaching losses were attributed to limitations in water content and the effect of HLRs on N transformation rates.

Climate change may produce considerable variations to rainfall rates, sea level and temperatures in the U.S., including the Northeast [31]. These climatic conditions are expected to affect the performance of OWTS and subsequently N transformations in the STA because nitrification and denitrification are sensitive to flow and water content, as well as temperature.

A limited number of studies have investigated the N fate, transport and removal mechanisms of N in STAs receiving ATE [32–35]. None of these studies has numerically modeled N transformations in STAs dosed with ATE. Little is known about nitrification and denitrification rates in advanced STAs, and no modeling approach has been developed to simulate these transformation processes. In this study, we addressed this knowledge gap with a calibrated HYDRUS 2D [29] model using soil moisture content and N speciation data collected from a conventional P&S drainfield and two types of shallow narrow drainfields (PSND and Geomat, a type of PSND) using replicated (*n = 3*) intact soil core mesocosms. We determined nitrification and denitrification rate coefficients for the three drainfield types and used these data to estimate N losses. The simulated results were compared to actual experimental data. The calibrated model was then used to predict the effect of climate changing conditions (increased temperature and rising water table) on nitrification and denitrification rate transformation in the drainfields. The information obtained from these models is expected to aide designers of OWTS and regulators to make informed decisions about the most effective treatment practices for removal of N species in the STA.

## Material and Methods

### Experimental Setup

Replicated mesocosms (*n = 3*) were engineered to mimic the soil treatment area and wastewater delivery system of a PSND, Geomat, and P&S. All systems were maintained at 20°C ± 0.7 with a water table separated 90 cm (PSND and Geomat) or 30 cm (P&S) from the infiltrative surface, representative of current climate conditions [20]. Mesocosms consisted of polyvinyl chloride (PVC) pipes (0.15 m ID, 1.5 m H) containing undisturbed soil that is representative of the soil profile used for an STA of an OWTS in southern New England (Morphological, physical and chemical properties of the soil are listed in S1 Table).

Mesocosms were dosed with domestic wastewater, based on accepted guidelines for frequency and volume of wastewater inputs for the State of Rhode Island. For P&S mesocosms, STE was applied at a rate of 400 mL d^-1^ in two doses of 200 mL over 1.5 h every 12 hours. PSND and GEO mesocosms were dosed with ATE at a rate of 2 L d^-1^, in 42-mL doses over 15 min every 30 min. The wastewater was dispersed 20 cm below ground surface for PSND, at 25 cm for GEO, and at 84 cm for P&S. The mesocosms were instrumented with probes to collect soil moisture and temperature data.

Effluent samples, along with wastewater inputs, were analyzed weekly for total N, ammonium and nitrate, and other water quality parameters. The physical, chemical and microbiological characteristics of STE and ATE are summarized in S2 Table. Detailed information about soil mesocosms setup and water analysis methodology are found in Cooper et al. [20].

### Modeling Approach

HYDRUS 2D/3D version 2.0 was used to simulate water flow and solute transport in soils under variably-saturated conditions. The HYDRUS program numerically solves the Richards equation for saturated-unsaturated water flow (Eq 1):

$$\frac{\partial \text{θ}}{\partial \text{t}}=\frac{\partial }{\partial {\text{x}}_{\text{i}}}\left[\text{K}\left({\text{K}}_{\text{ij}}^{\text{A}}\frac{\partial \text{h}}{\partial {\text{x}}_{\text{j}}}+{\text{K}}_{\text{iz}}^{\text{A}}\right)\right]-\text{S}$$ (1)

where θ is the volumetric water content [L^3^L^-3^], h is the pressure head [L], S is a sink term [T^-1^], x_i_ and x_j_ (i and j = 1,2) are the spatial coordinates [L], t is time [T], K_ij_^A^ are components of a dimensionless anisotropy tensor K^*A*^, and K is the unsaturated hydraulic conductivity function [LT^-1^] given by

$$K\left(h,x,y,z\right)=K_{s}\left(x,y,z\right)\;K_{r}\left(h,x,y,z\right)$$ (2)

where K_r_ is the relative hydraulic conductivity and K_s_ the saturated hydraulic conductivity [LT^-1^].

HYDRUS allows the user to select among several analytical models to describe the soil water retention and unsaturated hydraulic conductivity functions. In our model, the van Genuchten [36] equation was applied to compute the soil hydraulic properties (Eqs 3–5):

$$\theta \left(h\right)=\theta_{r}+\frac{\theta_{s}-\theta_{r}}{{\left[1+{\left|-\alpha h\right|}^{n}\right]}^{m}}$$ (3)

where *α* (L^-1^), *m* (dimensionless), and *n* (dimensionless) are fitted parameters, *θ*(*h*) is the volumetric water content (L^3^ L^-3^), *θ*_*s*_ is the saturated volumetric water content (L^3^ L^-3^), and *θ*_*r*_ is the residual volumetric water content (L^3^ L^-3^). The unsaturated hydraulic conductivity function *K(h)* (LT^-1^) is written as follows:

$$K\left(h\right)=K_{s}S_{e}^{l}{\left[1-{\left(1-S_{e}^{\frac{1}{m}}\right)}^{m}\right]}^{2}$$ (4)

$$S_{e}=\frac{\theta -\theta_{r}}{\theta_{s}-\theta_{r}}$$ (5)

where *m = 1-(1/n)* and *l* is the pore connectivity parameter, which it is assumed to be about 0.5 [37]. The model permits the application of the convection—dispersion equation in the liquid phase to simulate solute transport and fate. Chemical equilibrium and linear adsorption is described by the following mass balance equation:

$$\frac{\partial \theta \text{c}}{\partial \text{t}}+\rho {\text{K}}_{\text{d}}\frac{\partial \text{c}}{\partial \text{t}}=\frac{\partial }{\partial \text{x}}\left(\theta {\text{D}}_{\text{ij}}^{\text{w}}\frac{\partial \text{c}}{\partial {\text{x}}_{\text{j}}}\right)+\frac{\partial }{\partial \text{z}}\left(\theta {\text{D}}_{\text{ij}}^{\text{w}}\frac{\partial \text{c}}{\partial {\text{z}}_{\text{j}}}\right)-\frac{\partial {\text{q}}_{\text{x}}\text{c}}{\partial \text{x}}-\frac{\partial {\text{q}}_{\text{z}}\text{c}}{\partial \text{z}}-\mu \theta \text{c}$$ (6)

where *c* is dissolved solution concentration [ML^−3^], t is time (T), K_d_ is the adsorption coefficient (L^3^M^-1^), *μ* represents the solute transformation or degradation rate in the liquid phase, *x* is the solute travel distance (L) and *z* is depth (L). D^w^_ij_ is the dispersion coefficient tensor for the liquid phase [L^2^T^−1^], *θ* is the volumetric water content [L^3^L^−3^], *ρ* is the bulk density of porous medium [ML^−3^], and *q*_*x*_ and *q*_*z*_ is the specific discharge [LT^−1^] along the horizontal and vertical direction, respectively.

### Model Domain and Boundary Conditions

**Fig 1:**
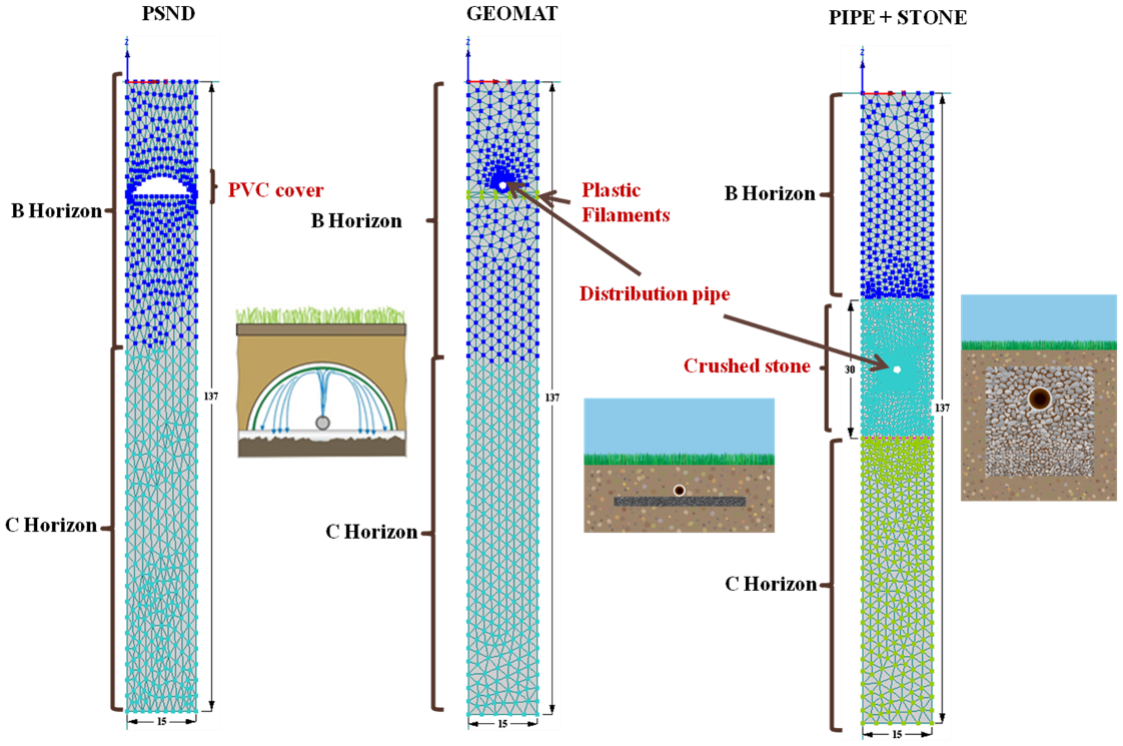
Model domain and porous material distribution for pressurized shallow narrow (PSND), Geomat (GEO), and pipe and stone (P&S) drainfield mesocosms. All dimensions are in cm.

The model domain was developed to resemble the mesocosms, not only physically but also in terms of operational conditions. The geometry of the domain properties reproduced the two shallow and trench drainfields described previously [20]. The model domain consisted of a 2D vertical plane (x-z) (rectangular, L = 15 cm, H = 137 cm high) (Fig 1).

The infiltrative surface was placed below the top boundary that shaped the ground surface. The PSND system consists of lateral pipes that distribute the advanced-treated effluent by squirting it against a cover made of larger diameter pipe cut longwise. It was modeled by an arc that represents an impermeable half-pipe cover located above the drainfield. The GEO system is comprised of a core of entangled plastic filaments and a pressure distribution pipe covered with a protective layer of geotextile fabric. GEO was modeled by including a 1-cm filament core layer and a 2.54-cm diameter circle on the top, which simulates the distribution pipe. The P&S model integrates a 30-cm layer (crushed stone or gravel backfill) with an embedded 2.54-cm diameter circle of simulated perforated pipe located 60 cm below the soil surface.

The native soil in the mesocosms is a Bridgehampton silt loam (coarse-silty, mixed, active, mesic Typic Dysturdept) (S1 Table). As mentioned previously, the infiltrative surface was placed 20–25 cm below the ground surface for PSND and GEO (A horizon), and 84 cm (C horizon) for P&S. Based on field observations, two layers were used to simulate B (gravelly loamy sand) and C (gravelly coarse sand, 40–45% gravel) horizons. For the purpose of this study and because of their similarities in the particle size distribution, sublayers B_w_ and 2B_w_ were modeled as one single layer.

A finite element mesh with a maximum element size of 3.90 cm was generated automatically with 478, 537 and 614 nodes for P&S, GEO and PSND, respectively. A denser grid was defined around the simulated distributed pipes and the PVC cover. Element size in that area was 0.45 cm. Observation nodes were located along the soil profile to compare the observed against modeled data. Two observation nodes were placed 15 cm and 30 cm below the infiltrative surface, a third node located at the bottom of the model domain and a fourth node at the column outlet.

**Fig 2:**
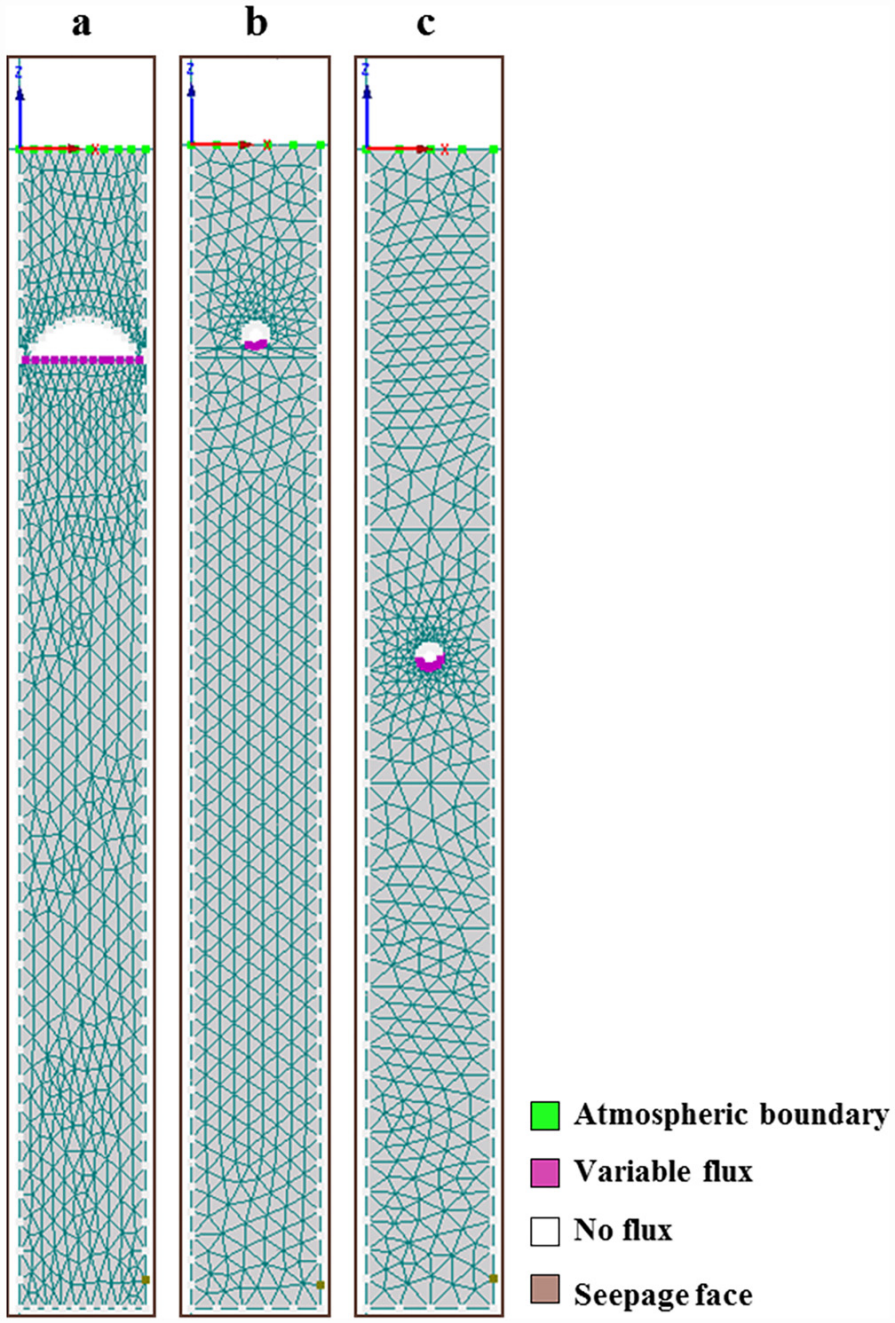
Boundary conditions for (a) pressurized shallow narrow (PSND), (b) Geomat (GEO) and, (c) Pipe and stone (P&S) soil drainfield mesocosms.

Atmospheric boundary condition was assigned to the top of the columns (Fig 2). The sides and bottom of the column were treated as no-flux boundaries. As wastewater infiltrates, it accumulates at the bottom and flows out when the soil is saturated or a hanging water table is formed. In order to account for this condition, a seepage face boundary was selected for one of the nodes at the bottom side of each soil column, as indicated in Fig 2. In the HYDRUS program, this occurs when the water is removed by overland flow once saturated conditions prevail [29].

### N Transformation Modeling

Nitrogen losses in STA are attributed to NH_4_^+^ conversion to NO_3_^-^ or nitrification followed by reduction of NO_3_^-^ to N_2_O or N_2_ through denitrification. Nitrification proceeds in a two-step reaction. First, NH_4_^+^ is oxidized to NO_2_^-^. Although not specifically investigated, this reaction is assumed to be catalyzed by bacteria through (Eq 7):

$${\text{NH}}_{4}^{+}+\;1.5{\text{O}}_{2}\overset{}{\to }{\text{ NO}}_{2}^{-}+{\text{H}}_{2}\text{O}+2{\text{H}}^{+}$$ (7)

Secondly, NO_2_^-^ is assumed to be oxidized to NO_3_^-^ by bacteria as follows (Eq 8):

$${\text{NO}}_{2}^{-}+\;0.5{\text{O}}_{2}\overset{}{\to }{\text{ NO}}_{3}^{-}$$ (8)

Denitrification is the transformation of NO_3_^-^ to its gaseous form (N_2_) and represents of one of the main mechanism of nitrogen loss in soils. N_2_ is produced from the reduction of NO_3_^-^ by heterotrophic-denitrifying bacteria. Denitrification takes place under anaerobic conditions and requires organic carbon (C). NO_3_^-^ is reduced to N_2_ through electron transfer from the organic C by the reaction:

$${\text{C}}_{6}{\text{H}}_{12}{\text{O}}_{6}+4{\text{NO}}_{3}^{-}\;\overset{}{\to }6{\text{CO}}_{2}+6{\text{H}}_{2}\text{O}+2{\text{N}}_{2}$$ (9)

We developed a decay model to simulate the fate and transport of N species in conventional and advanced STAs in which N was assumed to be transformed as follows [22]:

$${\text{NH}}_{4}^{+}\;\to {\text{ NO}}_{3}^{-}\;\to {\text{ N}}_{2}$$ (10)

Nitrite-oxidizing bacteria typically react much faster than NO_2_-producing bacteria, which restrict the accumulation of NO_2_^-^ [38–41]. For this reason we assumed that NH_4_^+^ is converted directly to NO_3_^-^, i.e. the production of NO_2_^-^ (Eq 8) was excluded from the process. From Eq 7, the rate of change of nitrification and denitrification can be expressed as:

$$\frac{\partial [\text{N}{\text{O}}_{3}^{-}]}{\partial \text{t}}=-\frac{\partial [\text{N}{\text{H}}_{4}^{+}]}{\partial \text{t}}=\mu_{\text{nit}}$$ (11)

$$\frac{\partial [{\text{N}}_{2}]}{\partial \text{t}}=-\frac{\partial [\text{N}{\text{O}}_{3}^{-}]}{\partial \text{t}}=\mu_{\text{Denit}}$$ (12)

where *μ*_*nit*_ and *μ*_*denit*_ are described as nitrification and denitrification reaction rates, respectively. N species were assumed to be dissolved in the STE and ATE and thus the nitrification and denitrification rate coefficients were only determined for the aqueous phase. Nitrogen species were modeled using sequential decay reactions built into HYDRUS [42]. The program provides nonlinear non-equilibrium reactions (adsorption-desorption) between the solid and liquid phases (soil-water interface) based on the two-site sorption concept [43,44]. It assumes that the sorption sites are composed of two fractions; sorption to one fraction of sites is assumed to be instantaneous, while for the remaining sites sorption is time-dependent. Also, it assumes that the solute transport in the bulk of the pore space takes place by convection and dispersion. The measured total N (TN) was modeled as an input concentration to include all N infiltrated into the drainfield. The influent organic N was considered to be mineralized to NH_4_^+^ through microbial decomposition:

$${\text{N}}_{\text{Org}}\overset{\text{Bacteria}}{\to }{\text{N}}_{\text{Min}}$$ (13)

Several researchers have reported that nitrification and denitrification processes are dependent on the water content [45,46]. Nitrification is an aerobic process that occurs at low soil water content because high soil water content increases tortuosity and, limits oxygen diffusion and the activity of nitrifying bacteria [47]. Denitrification takes place under saturated soil conditions, which promotes anoxia. HYDRUS was modified to account for the effect of soil water content on N transformation. For this, a water content dependency function in the DRAINMOD-N2 [48] module allows for computing nitrification and denitrification rates at low water saturation or unsaturated conditions based on Michaelis-Menten kinetics [49]. Both processes are modeled as first-order kinetics when the substrate concentration (NH_4_^+^and NO_3_^-^) is limited. The reaction kinetics change into zero-order reaction when substrate concentration increase [50]. Because the three drainfield types receive STE and ATE containing high concentrations of NH_4_^+^ that are rapidly converted to NO_3_^-^, nitrification and denitrification were modeled as zero-order kinetics. For nitrification the model uses a stepwise function to simulate the influence of nitrification inhibitors on decay rates. The following expression describes the nitrification rate:

$$\mu_{nit}=\mu_{nit,max}\left(\frac{C_{NH_{4}}}{K_{m,NH_{4}}+C_{NH_{4}}}\right)f_{t}f_{sw}$$ (14)

where *μ*_*nit*_ is the calculated nitrification rate (ML^-1^T^-1^), *μ*_*nit*,max_ is the maximum nitrification rate (MM^-1^T^-1^), *C*_*NH4*_ is the NH_4_^+^-N concentration (MM^-3^), and *K*_*m*,*NH4*_ is the NH_4_^+^-N half-saturation constant (MM^-1^T^-1^), which is the NH_4_^+^-N concentration at which the nitrification rate is half its maximum value. The parameter *f*_*sw*_ is the water content dependency function and is written as follows (Eq 15):

$$\begin{array}{l} f_{sw}=\{\begin{array}{ll} f_{s}+(1-f_{s}{\left(\frac{1-S}{1-S_{h}}\right)}^{e_{1}} & S_{h}<S\leq 1\\ f_{wp}+\left(1-f_{wp}\right){\left(\frac{1-S_{wp}}{S_{l}-S_{wp}}\right)}^{e_{2}} & S_{wp}<S\leq S_{l} \end{array} \end{array}$$ (15)

where *fsw* varies between 0 and 1. The term *fs* is the value of *fsw* at full saturation, *fwp* is the value of *fsw* at the wilting point, *Sh* and *S*_*l*_ are the upper and lower saturation boundary for optimal nitrification, *swp* is the saturation level at the wilting point, *S* is the actual soil saturation or water-filled pore space, and *e*_*1*_ and *e*_*2*_ are fitting parameters.

Denitrification is modeled as a function of the NO_3_^-^ concentration available and the organic content decrease with depth [41]. The denitrification rate equation included in the modified HYDRUS version is:

$$\mu_{denit}=\mu_{denit,max}\left(\frac{C_{NO_{3}}}{K_{m,NO_{3}}+C_{NO_{3}}}\right)f_{t}f_{sw,dn}f_{z}$$ (16)

where *μ*_*denit*_ is the denitrification rate (ML^-1^T^-1^), *μ*_*denit*,*max*_ is the maximum denitrification rate (MM^-1^T^-1^), *C*_*NO3*_ is the NO_3_^-^-N concentration, and *K*_*m*,*NO3*_ is the NO_3_^-^-N half-saturation constant (MM^-1^T^-1^), which is the concentration at which the denitrification rate is half its maximum value. The terms *f*_*t*_ and *f*_*z*_ are temperature-dependency and carbon dependency functions, respectively.

$$f_{t}=exp\left[-0.5\beta T_{opt}+\beta T\left(1-\frac{0.5T}{T_{opt}}\right)\right]$$ (17)

$$f_{z}=e^{-\alpha z}$$ (18)

$$f_{sw,\;dn}=\{\begin{array}{ll} 0 & S<S_{dn}\\ {\left(\frac{S-S_{dn}}{1-S_{dn}}\right)}^{f} & S\geq S_{dn} \end{array}$$ (19)

*ft* varies between 0 and 1. *T* is the temperature, *Topt* is the optimum temperature for nitrification, which is a system property and typically ranges from 20°C to 25°C, *β* and *α* are fitting parameters, and *z* is depth below the infiltrative surface. The term *f*_*sw*,*dn*_ is the water content-dependency function at a threshold saturation value for denitrification *(s*_*dn*_), *s* is the actual soil saturation, and *f* is a fitting exponent.

### Climate Change Simulation

Climate projections indicate that precipitation, sea level and temperatures have been increasing in parts of the United States, and this tendency is expected to continue [31]. Increased precipitation and sea level rise may lead to rising water table and a reduced treatment depth, which affect the performance of OWTS, particularly in coastal areas. Warmer soil temperatures are likely to affect N transformations in the drainfield because nitrification and denitrification rates are sensitive to soil temperature and water content, which might restrict the production of NO_3_^-^ and subsequent transformation to N_2_ [26]. To predict the response of N transformations, and the fate and transport of N in the three drainfield types to changing climate conditions, the calibrated model was used to simulate nitrification and denitrification under three scenarios: (i) warmer soil temperature (23°C vs. 20°C), (ii) rising water table, where the thickness of the unsaturated zone inside the mesocosms was reduced by 30 cm (from 112 cm to 82 cm), and (iii) simultaneously rising soil temperature and water table elevation. Observation nodes were located at the water table level and the column bottom outlet to record the modeled N species concentrations (only for scenarios ii and iii). The changing climate conditions were set based on climatic projections of eastern U.S. that suggest the water table and temperature will increase about a foot (30 cm) and ±3°C, respectively [31].

### Solute Transport Parameters

The transport characteristics were estimated for each of the soil drainfield mesocoms. The longitudinal dispersivity (λ_L_) was set to be one-tenth of the soil profile beneath the infiltrative surface [51,52]. A similar approach was used by others [53,54] to model nitrification and denitrification of septic tank effluent using HYDRUS 2D. Soil bulk density was measured for the silt loam and gravelly-coarse sand (S1 Table). A bulk density value of 1.50 g cm^-3^ was assigned to the entangled plastic filaments and crushed stone. The diffusion coefficients for NH_4_^+^ and NO_3_^-^ were set to 0.067 and 0.061 cm^2^ h^-1^, respectively [53,55].

### Calibration and Parameter Sensitivity

Model calibration was carried out to determine input parameter values for obtaining the best fit between the predicted and measured soil data. The model was calibrated by coupling HYDRUS with UCODE, a computer program used to estimate parameters through inverse modeling by nonlinear regression [56]. The nonlinear regression problem was solved by minimizing a weighted least-squares objective function with respect to the parameter values using a modified Gauss-Newton method.

A sensitivity analysis in UCODE was performed to identify which of the parameters influenced the model output results and their uniqueness. Composite scaled sensitivities (CSSs) were calculated to identify the influence of the observed data on the estimation of a parameter. CSS is a measure of the total amount of information provided by the observations to estimate one parameter. Larger CSS values indicate that those parameters are likely to be estimated more precisely with the proposed model and observations. The ratio of the CSS of a parameter to the maximum CSS was used to compare relative sensitivity among estimated parameters. Parameters with CSS ratio less than 0.01 are not sensitive and denote that a regression will not converge. Therefore, in some cases, parameters with CSS ratio < 0.01 were excluded from the inverse modeling process.

The model was calibrated by fitting water content and nitrogen species data (NH_4_^+^ and NO_3_^-^ concentration). HYDRUS water flow and solute transport modules were applied to complete the calibration. First, water content data were fitted to obtain the soil hydraulic parameters and evaluate the impact of moisture content on N transformation. Secondly, NH_4_^+^ and NO_3_^-^ concentration data were used to determine the nitrification and denitrification rates, and to estimate N losses.

The model was initially run under near saturation conditions to reach steady water flow in a shorter simulation time. Therefore, initial average pressure heads were set to -50 cm for the entire model soil profile. Atmospheric boundary conditions were assigned to the top of the model domain or simulated soil surfaces. The minimum permissible pressure was assumed to be -1,000 cm. No precipitation, evapotranspiration or root uptake was included in the simulated N transformation.

Hydraulic loading rates were modeled by assigning a variable flux boundary condition in each of the soil mesocosms. For PSND, it was assumed that wastewater was distributed uniformly over the entire infiltrative surface. For GEO and P&S, the variable flux boundary was located below the distribution pipe. SFE and STE deliveries were modeled as applied in the mesocosm experiments.

Initial values for soil hydraulic parameters were determined by Rosetta [57], a computer program that is part of HYDRUS. The software estimates soil water retention by implementing hierarchical pedotransfer functions (PTFs) based on soil textural classes. Fitting parameter values were assigned to the entangled plastic filaments (GEO) and crushed stone (P&S) systems. Both materials were considered highly conductive (K_s_ = 3,000 cm day^-1^), with low porosity and residual water content that was similar to a coarse gravel soil. Initial parameter values for native soils were estimated using Rosetta [57] and fitted with UCODE, whereas values for the plastic filaments and gravel layers were kept fixed. Initial N transformation rates were selected from McCray [22] and initial NH_4_^+^ and NO_3_^-^ soil concentration were set to zero. Water dependency function parameters were selected from McCray et al. [58]. Finally, the model was run for a 3-month (90 days) simulation period. The predicted N species concentrations were computed to estimate the N balance produced by each of the three OWTS. The best fit between the predicted and observed data was evaluated based on the root mean squared error (RMSE). A RMSE value closer to zero indicates the best of fit to observed data.

## Results and Discussion

### Water Content

The model was calibrated using soil moisture data to simulate the unsaturated soil profile beneath the infiltrative surface and to account for moisture changes associated with N transformation processes. Given that variations in water content around the measured moisture data were minimal, the mesocoms simulations were found to be under steady-state conditions. The soil hydraulic parameters (*θ*_*r*,_ *θ*_*s*,_ *α*, K_s_, *n* and *l*) were determined for each of soil layer (silt loam and gravelly-coarse sand); only the pore connectivity parameter value was not calibrated or changed (*l* was equal to 0.5, as recommended by [29]). PSND and GEO, five soil hydraulic parameters (*θ*_*r*_, *θ*_*s*_, *α*, K_s_ and *n*) for each of the two horizons (silt loam and gravelly-coarse sand) were calibrated simultaneously (10 parameters total). Five parameters were used for the conventional P&S (Table 1).

**Table 1:** Model soil hydraulic parameters calibrated in advanced and conventional drainfield mesocosms. The parameters with subscripts "s" and "g" denote the set of parameters for silt loam and gravelly-coarse sand soils, respectively.

	Symbol	
Parameter	Silt loam	Gravelly—coarse sand
Residual water content	*θ*_*rs*_	*θ*_*rg*_
Saturated water content	*θ*_*ss*_	*θ*_*sg*_
Pore-size distribution index	*n*_*s*_	*n*_*g*_
Saturated Hydraulic conductivity	*K*_*ss*_	*K*_*sg*_
Inverse of the air-entry value	*α*_*s*_	*α*_*g*_

**Fig 3:**
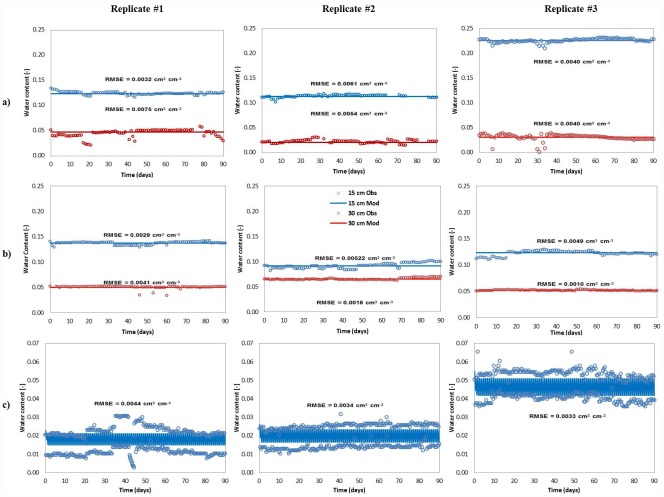
Observed and simulated water content for (a) pressurized shallow narrow (PSND), (b) Geomat (GEO) and (c) Pipe and stone (P&S) drainfield mesocosms. Root mean square error (RMSE) is included as a measure of the goodness-of-fit between predicted and observed data.

In PSND and GEO, the measured water content (cm^-3^cm^-3^) ranged from 0.11 to 0.13 and 0.02 to 0.05 at 15 cm and 30 cm below the infiltrative surface, respectively. Even though the intact soil cores were collected in close proximity to each other, water content variations were expected at greater depths because of the cumulative influence of variable physical properties on soil moisture and water flow with depth. Also, the amount of water retained in the upper soil layer was expected to affect the hydraulic properties of the deeper soil layers. This heterogeneous behavior of the soil system is illustrated by the three PSND mesocosms, i.e. one mesocosm showed higher water content (0.23 cm^-3^cm^-3^) at the 15-cm depth compared to the other two (0.11 to 0.13 cm^-3^cm^-3^). In general, these variations are indicative of soils with low residual and high saturated water content characteristics (Fig 3).

Overall, the model resulted in a good fit between the observed and simulated water content data for PSND, GEO and P&S (Fig 3). RMSE values for PSND and GEO ranged from 0.0010 and 0.0075 cm^-3^cm^-3^ for silt loam and gravelly-coarse sand, indicating good agreement between the simulated and measured data. The goodness-of-fit is illustrated in Fig 3, where the model output data were described by a straight line indicating steady conditions during the entire period of simulation at both observation nodes (15-cm and 30-cm depths). In case of the P&S mesocosms, these were dosed with wastewater every 12 h, which resulted in a comparatively drier soil profile and longer times of unsaturated flow between doses relative to the PSND and GEO dosing scheme. Thus, variations in soil moisture content were observed between dosing events, with soil moisture values varying by a factor of two. The water content peaked immediately after dosing (0.03 cm^-3^cm^-3^ to 0.05 cm^-3^cm^-3^) and dropped quickly (0.01 cm^-3^cm^-3^ to 0.02 cm^-3^cm^-3^) between doses. Under steady state conditions, the model reproduced those fluctuations with acceptable goodness-of-fit (RMSE: 0.0033 cm^-3^cm^-3^ to 0.0044 cm^-3^cm^-3^).

**Fig 4:**
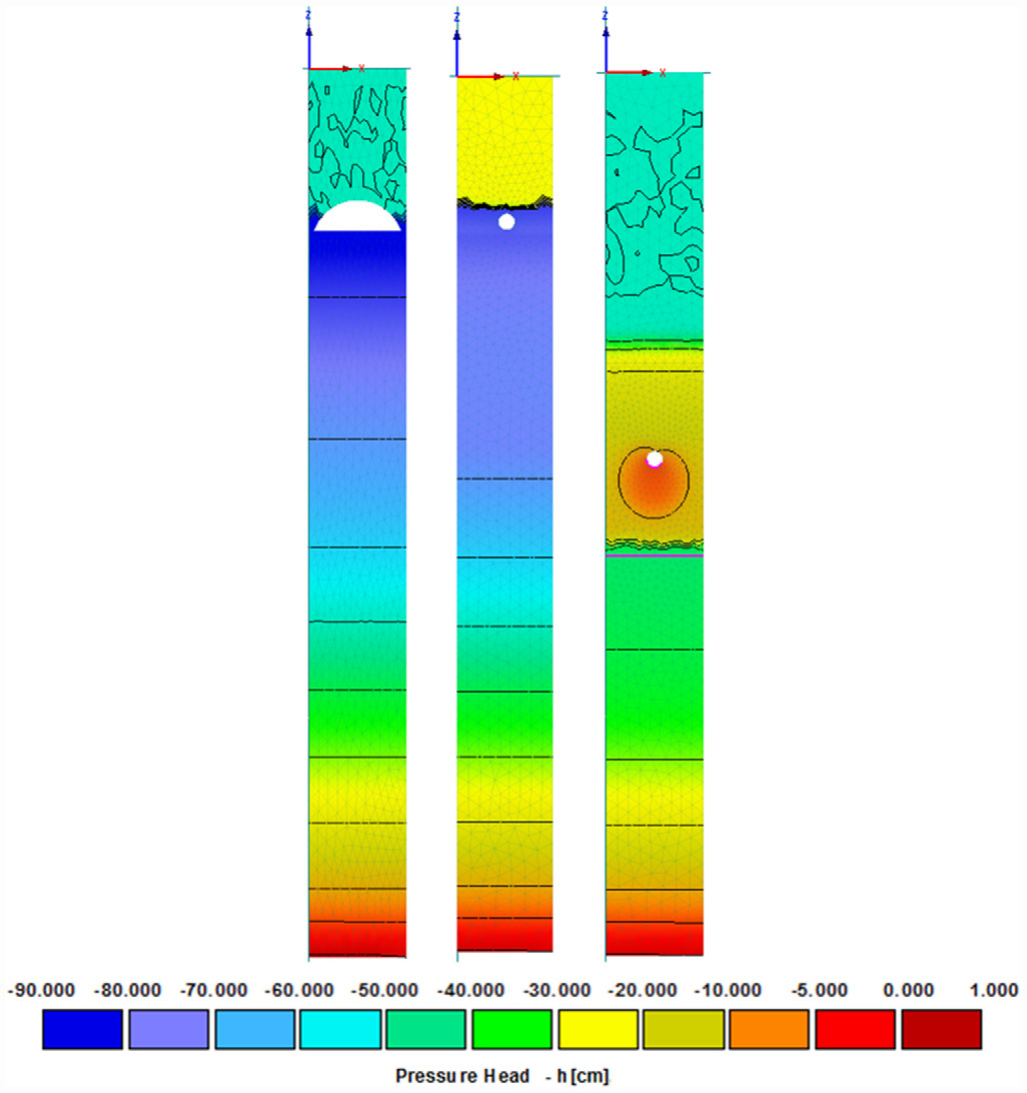
Pressure head distribution as a result of the seepage boundary condition to simulate a hanging water table at the bottom of the mesocosms. At steady state conditions, pressure head values are close to zero, which indicates that area is near or under saturation conditions.

The water content data were modeled under the effect of a simulated hanging water table at the bottom of the mesocosms, where the seepage face boundary (Fig 4) caused this part of the model domain to remain saturated once the system was at steady state. The calibrated retention curve parameters are shown in Table 2.

**Table 2:** Calibrated soil hydraulic parameters for the simulation of pressurized shallow narrow (PSND), Geomat (GEO) and pipe and stone (P&S) drainfield mesocosms. Values are means ± SD (n = 3).

Texture	Parameter	Units	PSND	GEO	P&S
	*θ*_*rs*_	cm^3^ cm^-3^	0.025 ± 0.002	0.024 ± 0.000	-
	*θ*_*ss*_	cm^3^ cm^-3^	0.203 ± 0.030	0.181 ± 0.017	-
**Silt loam**	*n*_*s*_	-	2.289 ± 0.590	2.282 ± 0.513	-
	*K*_*ss*_	cm day^-1^	220.02 ± 51.03	252.43 ± 19.43	-
	*α*_*s*_	-	0.0847 ± 0.097	0.0182 ± 0.003	-
	*θ*_*rg*_	cm^3^ cm^-3^	0.013 ± 0.001	0.014 ± 0.001	0.012 ± 0.001
	*θ*_*sg*_	cm^3^ cm^-3^	0.063 ± 0.034	0.138 ± 0.001	0.068 ± 0.034
**Gravelly to coarse sand**	*n*_*g*_	-	4.037 ± 0.412	4.282 ± 0.174	3.731 ± 0.687
	*K*_*sg*_	cm day^-1^	908.88 ± 26.82	942.48 ± 5.430	4.513 ± 0.19
	*α*_*g*_	-	0.0205 ± 0.005	0.0189 ± 0.001	0.0838 ± 0.0440

The calibrated values differed among soil layers, which indicate that the properties of the soil at the infiltrative surface were different from the underlying soil, likely due to differences in soil texture and structure. Based on the soil moisture data, the silt loam was less conductive and had higher saturated water contents. The underlying soil (gravelly-coarse sand) for the PSND and GEO was simulated with *K*_*sg*_ ranging from 908.9 to 942.5 cm day^-1^, which were 21% to 44% higher than reported values for sandy soil [59,60]. Although variations in hydraulic conductivity values are expected, in our study the higher *K*_*sg*_ were likely caused by the presence of significant amounts of gravel, which accounted for 40% to 45% of the soil by weight. These differences in physical properties affect the soil properties directly, and influence the hydraulic properties and water flow. The gravelly-coarse sand layer for the P&S drainfield mesocosms was simulated with an average hydraulic conductivity of 4.51 cm day^-1^ (Table 2). As indicated in Fig 4, it is most likely that a biomat developed overtime above the infiltrative surface, which results in unsaturated conditions and a reduced saturated hydraulic conductivity.

### Sensitivity Analysis

The sensitivity analysis was focused on five soil hydraulic parameters (*θ*_*r*,_ *θ*_*s*,_ *α*, K_s_ and *n*). The composite scale sensitivity ratios to the measured soil moisture data for the silt loam and gravelly-coarse sand soils for PSND, GEO and P&S are shown in Table 3. Most of the selected parameters were significantly sensitive (CSS ≥ 0.01) to the water content data. For the advanced STAs, the simulated soil moisture was sensitive to the silt loam soil properties. Not unexpectedly, the soil properties *θ*_*ss*_ and *n*_*s*_ were most important for the calibration of the hydraulic parameters along the soil profile. Generally, *K*_*ss*_, *θ*_*rs*_, *θ*_*rg*_ and *K*_g_ were either not significant or less sensitive parameters. For P&S, the saturated and residual water content (*θ*_*rg*_ and *θ*_*sg*_) were very important parameters determining the soil moisture distribution along the profile. In addition, the hydraulic conductivity (*K*_*sg*_) was more sensitive compared to PSND and GEO (CSS = 0.21 to 0.25).

**Table 3:** Composite scale sensitivity ratios to the measured soil moisture data for the silt loam and gravelly-coarse sand soils for pressurized shallow narrow (PSND), Geomat (GEO) and pipe and stone (P&S) drainfield mesocosms.

Parameter	PSND			GEO			P&S		
***θ***_***rs***_	0.08	0.09	0.01	0.06	0.14	0.08	-	-	-
***θ***_***ss***_	**1.00**	**1.00**	**1.00**	**1.00**	**0.96**	**1.00**	-	-	-
***n***_***s***_	0.32	**0.41**	**0.44**	**0.59**	**0.47**	**0.73**	-	-	-
***K***_***ss***_	0.09	0.13	0.08	0.08	0.10	0.12	-	-	-
***α***_***s***_	**0.43**	0.35	0.01	0.24	0.56	0.15	-	-	-
***θ***_***rg***_	0.08	0.08	0.02	0.07	0.10	0.08	**0.89**	**0.64**	0.17
***θ***_***sg***_	0.30	0.11	0.11	0.31	0.69	**0.38**	**1.00**	**1.00**	**1.00**
***n***_***g***_	0.22	0.08	0.02	0.26	0.44	0.31	**0.46**	**0.66**	**0.61**
***K***_***sg***_	0.07	0.04	0.00	0.06	0.06	0.06	0.24	0.21	**0.25**
***α***_***g***_	**0.39**	0.32	0.09	0.43	**1.00**	0.53	0.15	0.04	0.02

In one of the PSND columns (Table 3, column #3), the *K*_*sg*_ was found to be less sensitive to fitting water content data (CSS < 0.01). In this mesocosm, the water content of the silt loam was almost two times higher (0.23 cm^-3^cm^-3^) than in the other two PSND columns (0.11 cm^-3^cm^-3^ and 0.13 cm^-3^cm^-3^). These variations are likely linked to soil heterogeneity.

### Nitrogen Transport and Fate

Nitrification and denitrification were modeled using a water content-dependent function to account for changes in oxygen diffusion and its availability in the mesocosms. The function uses water-filled pore space to mimic soil aeration during water infiltration [61]. Based on this approach, NO_3_^-^ production is achieved with a water-filled pore space (WFPS) of 0.20, and the maximum nitrification rate is reached when WFPS is more than 0.35. Denitrification takes place when WFPS is more than 0.60 and the highest N_2_ gas production rate is observed at saturation (WFPS = 1.00) [62,63]. Linn and Doran [63] reported that organic carbon decomposition associated with N mineralization and immobilization occurs when WFPS ranges from 0.50 to 0.60 as well as saturation. Therefore, WFPS variation may affect the denitrification rates in the soil drainfield. However, it must be emphasized that the aqueous solution used in those experiments [62,63] had a higher dissolved oxygen concentration compared to the STE and ATE used in this study. Based on that, the relationship between WFPS and relative rate of microbial nitrification and denitrification may be affected during N transformation, and nitrification and denitrification may occur at lower WFPS than previously described.

The nitrification and denitrification rate coefficients were computed using Eqs 14 through 19, and parameter values were selected from literature data [58]. Also, the parameter values for the water-content dependent functions were fitted. Initially, the model was adjusted until the best fit was achieved between the observed and predicted data. As a result, the parameters for nitrification and denitrification dependency functions are median values that best reproduced the observed data (*f*_*wp*_ = 0, *f*_*s*_ = 0, *s*_*wp*_ = 0.154, *s*_*l*_ = 0.665, *s*_*h*_ = 0.809, *e*_*1*_ = 2.267, *e*_*2*_ = 1.104, *s*_*dn*_ = 0 and *f* = 2.86) [58].

The fitted water content was important to elucidate the N transformation and decay in the mesocosms and the application of the water-content dependent functions. The results showed that the WFPS was higher than 0.27 (P&S gravelly-coarse sand) in all drainfields types (Table 4).

**Table 4:** Modeled water-filled pore space (WFPS) for pressurized shallow narrow drainfield (PSND), Geomat (GEO) and pipe and stone (P&S) drainfield mesocosms. Values are means ± SD (n = 3).

	WFPS (cm^3^ cm^-3^)	
STA type	Silt loam	Gravelly-coarse sand
**PSND**	0.64 ± 0.06	0.41 ± 0.05
**GEO**	0.74 ± 0.15	0.56 ± 0.15
**P&S**	-	0.27 ± 0.02

This indicates that sufficient oxygen is available for nitrification to proceed. Compared to the gravelly-coarse sand, the silt loam material has the highest values for the modeled WFPS in both PSND and Geo (0.64 and 0.74, respectively). A similar value (0.76) was reported by Bradshaw et al. [26] when simulating nitrification and denitrification rates from an OWTS drainfield installed in a clay-textured soil using pressure head and NH_4_^+^and NO_3_^-^ concentration data to simulate the system. Their model converted the pressure heads into water content values to calculate the actual WFPS of the drainfield. It also captured the effect of seasonal changes (dry and wet weather) on N transformation and found that the computed WFPS was adequate for nitrification to occur.

Our results are consistent with what is expected for the soil types and the hydraulic properties of our mesocosm materials. The data indicate that nitrification occurred in the first few centimeters below the infiltrative surface. Nitrate production in all drainfields and at shallow depths (top 15 cm) is likely caused by the oxidation of ammonia by ammonia-oxidizing and nitrifying bacteria [20].

**Fig 5:**
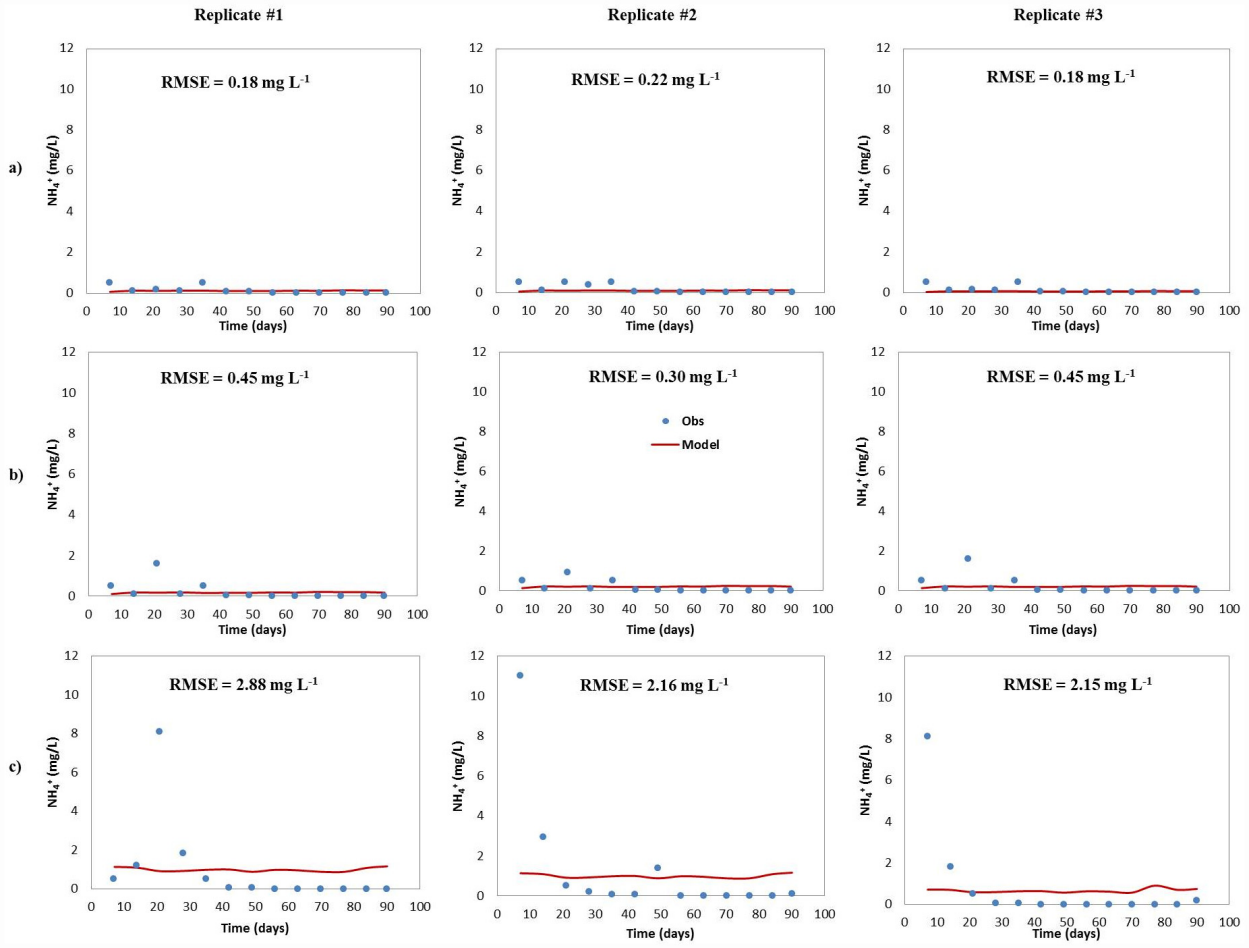
Predicted and measured NH_4_^+^ concentrations for (a) pressurized shallow narrow (PSND), (b) GEOMAT (GEO) and (c) pipe and stone (P&S) drainfield mesocosms.

The predicted and measured NH_4_^+^ concentrations for all drainfield types are shown in Fig 5. Except for the pipe and stone (P&S) drainfield mesocosm (Fig 5c), the model output show a good fit relative to the measured NH_4_^+^ concentrations in effluent water, with RMSE values ranging between 0.18 and 0.45 mg L^-1^. The P&S model resulted in higher RMSE values (2.18 to 2.88 mg L^-1^) because of initially elevated NH_4_^+^ concentrations. The cause for the elevated NH_4_^+^ concentrations is unknown and including these data caused the model to exceed the measured ammonia concentrations.

The maximum NH_4_^+^ concentration was found near the infiltrative surface (top 15 cm) and decreased with depth along the soil profile. The model results showed that the NH_4_^+^ was almost completely transformed at the 30-cm depth, consistent with other studies [25,64,65]. Moreover, the lowest measured and modeled NH_4_^+^ concentrations were observed in the outflow, where almost no NH_4_^+^ was detected.

**Fig 6:**
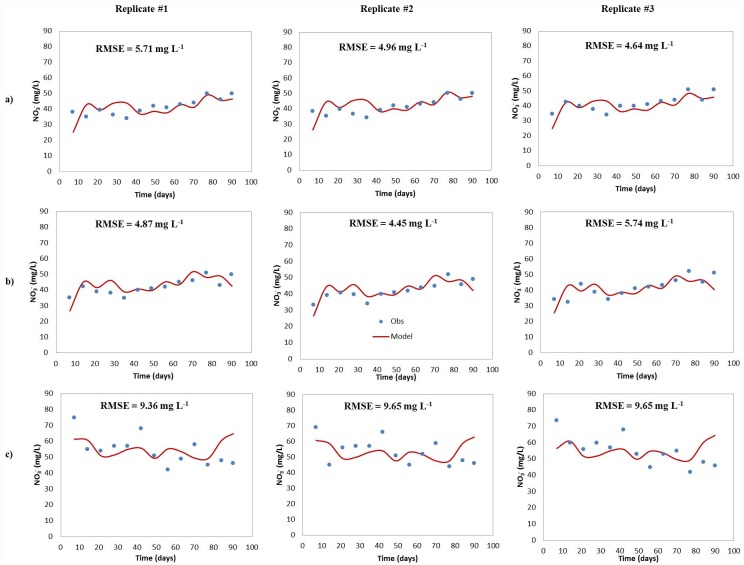
Predicted and measured NO_3_^-^ concentrations for (a) pressurized shallow narrow (PSND), (b) Geomat (GEO) and (c) pipe and stone (P&S) drainifield mesocosms.

The NO_3_^-^ concentration in ATE inputs and water exiting the mesocoms were measured and the data used to calibrate the models for the advanced and conventional STAs (Fig 6). In PSND and GEO, influent total N included NO_3_^-^ and NH_4_^+^. Some of the nitrate resulted from NH_4_^+^ being nitrified in the sand filter that preceded the treatment system from which the ATE was collected. Nitrate tended to increase with depth along the soil profile in all mesocosms, with the highest concentration detected near the outlet (seepage boundary).

For ATE, the model output included NO_3_^-^ initially present in the influent water as well as the NO_3_^-^ produced *in situ* from NH_4_^+^ oxidation. The model suggests that the remaining NH_4_^+^ will be transformed to NO_3_^-^ in the drainfield.

The predicted NO_3_^-^ concentrations showed an acceptable goodness-of-fit with the observed data, with RMSEs ranging from 4.45 mg L^-1^ d^-1^ to 9.65 mg L^-1^ d^-1^ in all STA types (Fig 6). Lower RMSE values were observed for PSND and GEO compared to P&S. This observation was not unexpected because ATE likely is more uniformly distributed over the infiltrative surface in the PSND and GEO.

### Nitrification and Denitrification Rates

Nitrogen transformation and removal is mainly controlled by nitrification and denitrification. In addition, NH_4_^+^ sorption to soil can affect the fate and transport of N in some OWTS drainfields. Because of the low sorption capacity of the soils studied herein (S1 Table), NH_4_^+^ sorption was not considered. Therefore, all NH_4_^+^ must move with the soil water and can be readily nitrified. Average simulated nitrification and denitrification zero-order reaction rates were computed to analyze the N dynamics and conversion in all drainfield types (Table 5).

**Table 5:** Average zero-order nitrification and denitrification rates for the selected soils and materials in pressurized shallow narrow (PSND), Geomat (GEO) and pipe and stone (P&S) drainfield mesocosms. Values are means ± SD (n = 3).

	Nitrification rates			Denitrification rates		
Material	PSND	GEO	P&S	PSND	GEO	P&S
	mg L^-1^ d^-1^					
**Silt loam**	45.25 ± 2.12	2.17 ± 0.09	-	0.17 ± 0.12	0.04 ± 0.01	-
**Gravelly-coarse sand**	49.19 ± 2.24	24.46 ± 1.06	3.83 ± 3.42	1.31 ± 0.96	0.31 ±0.10	0.36 ± 0.17
**Geomat**	-	25.88 ± 1.12	-	-	0.01 ± 0.00	-
**Crushed stone**	-	-	12.10 ± 3.72	-	-	0.44 ± 0.21

The nitrification rates ranged from 0.5 mg L^-1^ d^-1^ to 574 mg L^-1^ d^-1^ and were similar to zero-order rate values reported by McCray et al. [22]. Geza et al. [66] developed a tool for predicting the fate and transport of nitrogen in STAs (STUMOD), which uses nitrification rates as an input parameter. Their default value is 56 mg N L^-1^ d^-1^, which is similar to nitrification rates modeled herein. Overall, the advanced OWTS drainfields showed higher nitrification rates compared to P&S. As summarized in Table 5, the average PSND zero-order nitrification rates for silt loam and gravelly coarse sand were 45.25 mg N L^-1^ d^-1^ and 49.19 mg N L^-1^ d^-1^, respectively. Lower values were computed for GEO (2.17 mg N L^-1^ d^-1^ and 25.88 mg N L^-1^ d^-1^ for silt loam and gravelly-coarse sand, respectively). The model results suggest that some nitrification occurred in the entangled plastic filaments (25.88 mg N L^-1^ d^-1^). Nitrate production at that interface may be attributed to high oxygen diffusion and ATE aeration during the infiltration process. The average nitrification rates were 3.83 mg N L^-1^ d^-1^ in the gravelly coarse sand for the P&S. Nitrification took place at a rate of 12.10 mg N L^-1^ d^-1^ in the crushed stone and was 0.5 times lower than that computed for the GEO plastic filaments (25.88 mg N L^-1^ d^-1^). These data indicate that the presence of a conductive layer on the top of the infiltrating native soils provides an additional treatment zone for N removal. Furthermore, the higher NH_4_^+^ transformation rates in the advanced OWTS suggest that the drainfield placement at a shallower depth is more effective for nitrification than the conventional systems, likely because of a larger volume of unsaturated soil available for treatment.

Denitrification was not very significant in any of the OWTS drainfields. Denitrification rate values were one to three orders of magnitude lower than nitrification rates (from 0.01 to 0.44 mg N L^-1^ d^-1^). Tucholke et al. [67] reported higher zero-order denitrification rates values, ranging between 0.033 and 127 mg N L^-1^ d^-^1. However, those values [67] were obtained under fully saturated conditions (WFPS = 100%). Because unsaturated conditions prevailed in all mesocosms discussed herein, denitrification may have been restricted, since it requires anaerobic conditions to proceed [53]. Anaerobic conditions are more likely under saturated flow conditions.

Denitrification rates were higher in P&S than GEO and PSND. This finding is consistent with the experimental results presented in [20], where denitrification was higher in P&S compared to the other STAs. Besides anaerobic conditions, denitrification requires organic carbon to proceed [52]. Because ATE has low organic carbon content, it may have further limited the extent of denitrification in the advanced drainfield mesocosms. This would be consistent with [20].

### N Losses and comparison between Simulated and Real Systems

Average modeled N losses were calculated and compared with the experimental data from all of the advanced and conventional drainfield mesocoms. The calculations were based on the 90-day simulation period and accounted for all N species produced. An N mass balance was calculated from the modeled N species for influent and effluent water. In P&S, the modeled effluent N was comprised of dissolved NO_3_^-^ (82.72%) and NH_4_^+^ (1.41%). In GEO and PSND, the modeled effluent N speciation consisted of 89–91% NO_3_^-^ and 0.23–0.44% NH_4_^+^. The model results indicate that the total N losses as N_2_ were 10.44%, 9.65%, 17.60% for PSND, Geo and P&S, respectively. There were discrepancies between the computed and observed NO_3_^-^ data, particularly for the N removal in P&S. That is, the computed NO_3_^-^ data underestimated the measured data in some instances. It is likely that not all organic N was converted to NH_4_^+^ and as a result, less NH_4_^+^ was nitrified, whereas for our modeling approach, we assumed that organic N is completely transformed to NH_4_^+^ before entering the treatment system. Organic N accounted for 14% to 16% [20] of the total N in the effluent water in P&S, which is a significant amount for N loss. Also, a fraction of the influent organic N is likely non-biodegradable or recalcitrant (not amenable to ammonification), which means it might not be transformed in the treatment system and passing through the drainfield unchanged. For GEO and PSND, the modeled N losses occurred mostly as NO_3_^-^ (90.75% and 88.45%, respectively). Only minor percentages of NH_4_^+^ were observed during the 90-days simulation period (0.23 to 1.41% for all drainfield types). Nitrogen losses as N_2_ were more evident in P&S compared to the advanced technologies.

### Nitrification and Denitrification under Changing Climate Conditions

**Fig 7:**
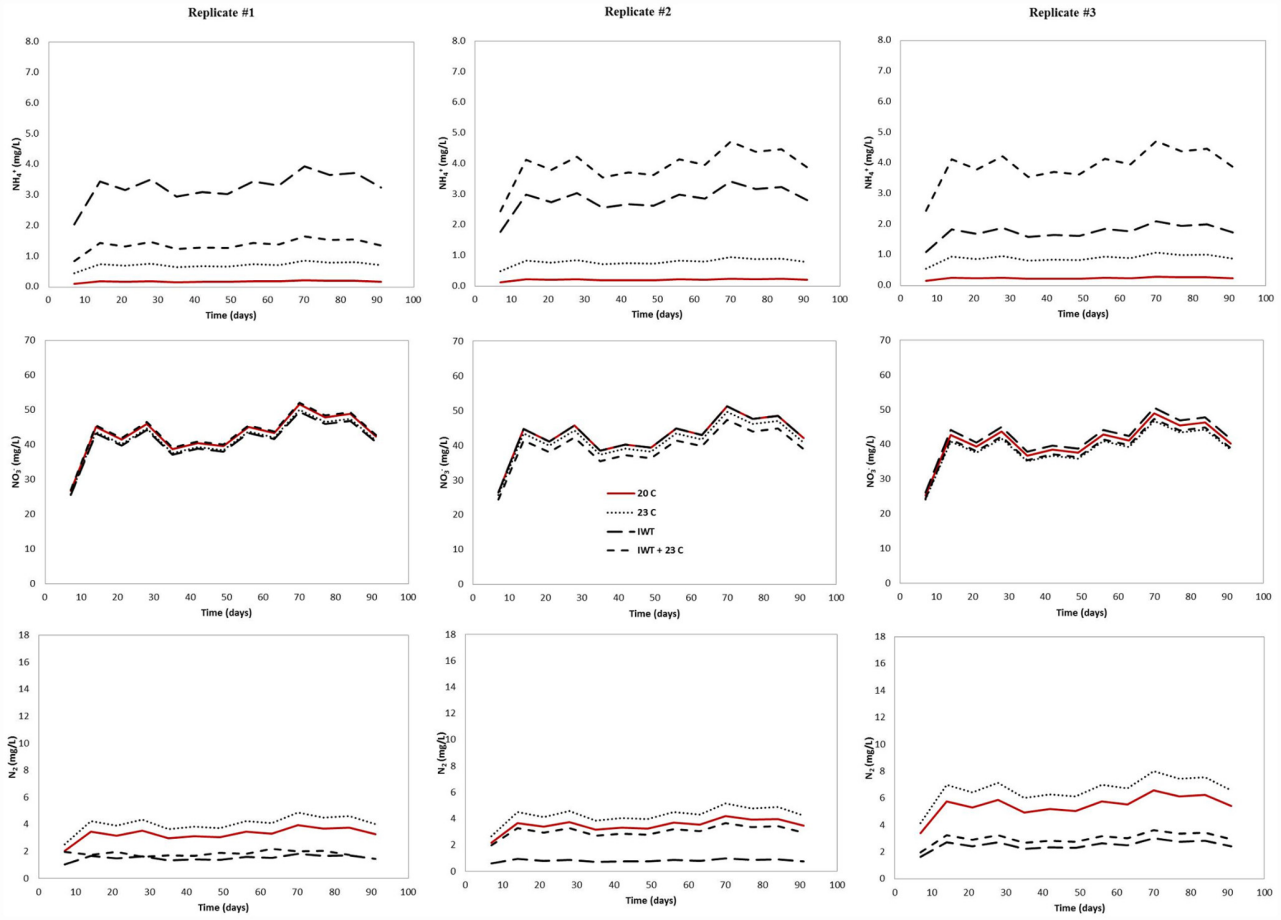
Predicted output NH_4_^+^, NO_3_^-^, and N_2_ concentration for pressurized shallow narrow drainfield (PSND) mesocosm under changing climate conditions. To evaluate the effect of climate change on soil treatment areas, the following scenarios were simulated: (i) warmer temperature (23°C), (ii) water table located at a shallower distance from the infiltrative surface (IWT) and (iii) a combination of both (IWT + 23°C).

**Fig 8:**
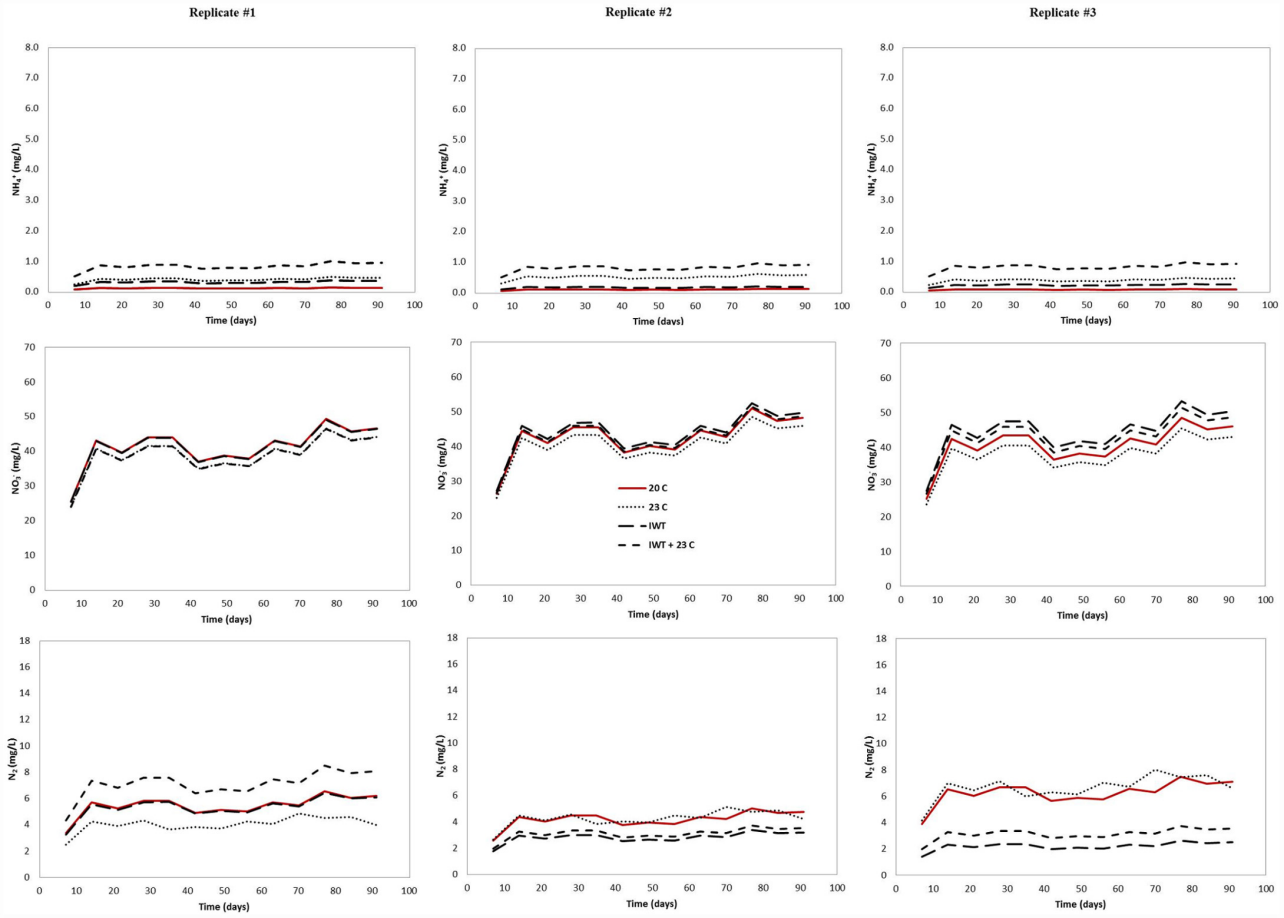
Predicted output NH_4_^+^, NO_3_^-^, and N_2_ concentrations for Geomat (GEO) drainfield mesocosm under climate changing conditions. To evaluate the effect of climate change on soil treatment areas, the following scenarios were simulated: (i) warmer temperature (23°C), (ii) water table located at a shallower distance from the infiltrative surface (IWT) and (iii) a combination of both (IWT + 23°C).

**Fig 9:**
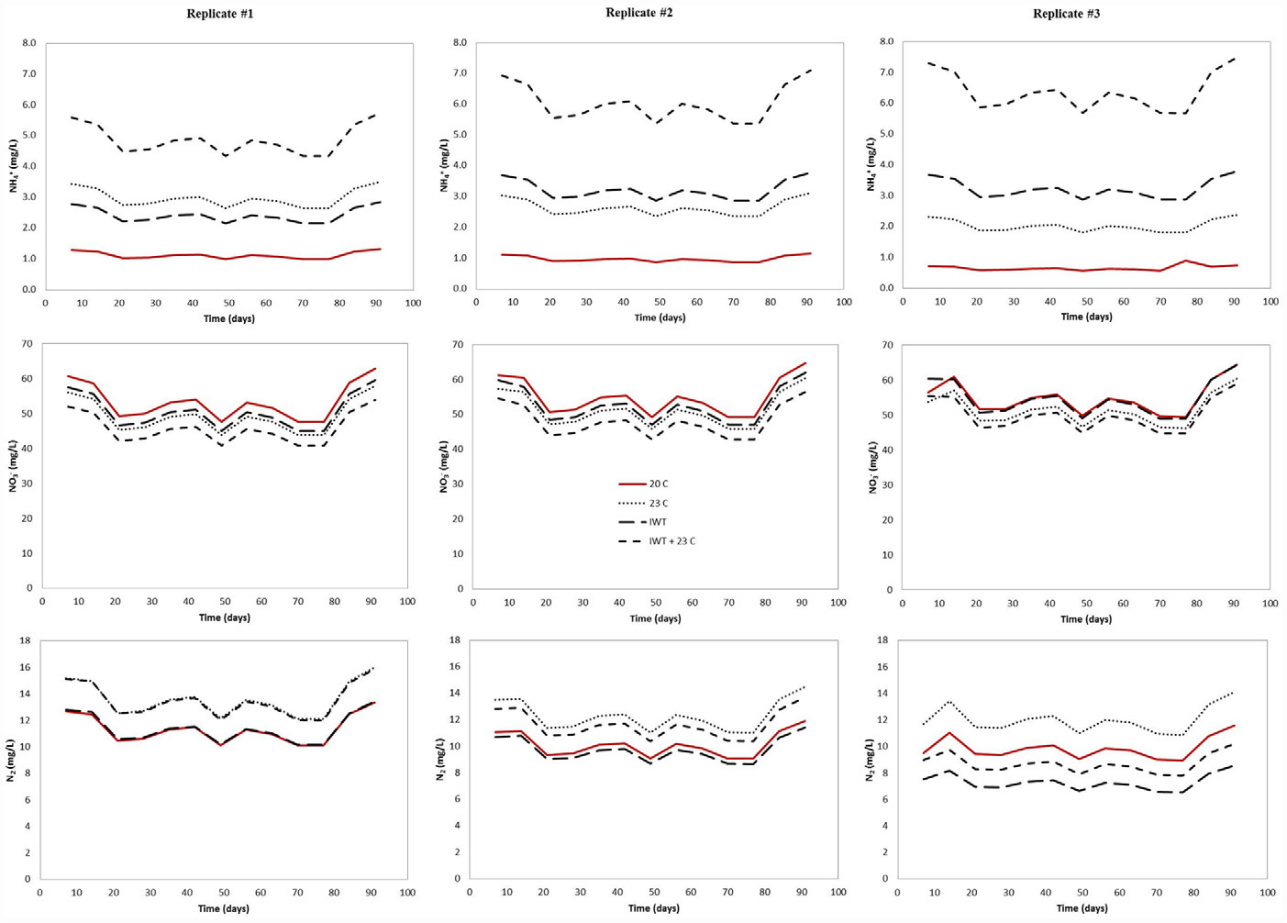
Predicted output NH_4_^+^, NO_3_^-^, and N_2_ concentration for pipe and stone (P&S) drainfield mesocosm under climate changing conditions. To evaluate the effect of climate change on soil treatment areas, the following scenarios were simulated: (i) warmer temperature (23°C), (ii) water table located at a shallower distance from the infiltrative surface (IWT) and (iii) a combination of both (IWT + 23°C).

Our model predicted the effect of warmer temperature and water table located at a shallower distance from the infiltrative surface on nitrification and denitrification in the three different drainfield type. The modeled NH_4_^+^ output concentrations were higher at the water table level in all simulated scenarios (Figs 7–9), indicating that nitrification was affected by the simulated environmental stresses, which resulted in less NH_4_^+^ converted to NO_3_^-^.

Ammonium output concentrations were more pronounced when the water table was raised and the soil temperature was increased, simultaneously. For example, the average NH_4_^+^ concentration was increased about one order of magnitude in the GEO (from 0.21 mg L^-1^ to 3.71 mg L^-1^) compared to those simulated at current conditions (soil temperature: 20°C and no rising water table). For PSND, when the water table was located at a shallower distance from the infiltrative surface and warmer soil temperature, only small differences were observed on average output NH_4_^+^ concentration, which was increased from 0.11 mg L^-1^ to 0.83 mg L^-1^. The greatest effect of climate changing conditions on nitrification was observed in P&S mesocosms, where the average effluent NH_4_^+^ concentration was about six times higher than under current conditions. Since N transformation was assumed to be the result of a sequential decay, NO_3_^-^ concentrations were also influenced by changing climate conditions. As a result, NO_3_^-^ concentrations decreased due to the effect of rising water table and warmer temperature. The relatively lower nitrification rate at the water table level can be attributed to a reduction in the amount of unsaturated soil, particularly the vertical separation between the infiltrative surface and the water table.

Denitrification was also restricted when our model for all drainfield types was run under climate changing conditions. Variations in N_2_ output concentration were observed in response to the differences in temperature and water table level. For instance, denitrification was most affected when the model was run with the water table located at shallower distance from the infiltrative surface and warmer soil temperature. Similar results were obtained when both conditions were modeled separately. In most mesocosms, N_2_ production increased with increasing temperature. At 23°C and current separation distance, N losses as N_2_ increased 22%, 37% and 21% for GEO, PSND and P&S, respectively. As the water table was located closer to the infiltrative surface, denitrification accounted for 100% of input NH_4_^+^ concentration at the saturation zone (column bottom) and NO_3_^-^ was not detected below the water table. This is attributed to the complete conversion of NO_3_^-^ to N_2_ in the saturated zone. The model incorporates a carbon dependency function to account for the effect of the spatial variability of C on denitrification rate (Eq 18) [61]. In this context, C availability is simulated as a function of soil depth (C content is decreased with depth in the soil profile). Therefore, denitrification was not expected below the water table due to carbon limitation. We assumed that the NO_3_^-^ plume would be likely transported and diffused rather than converted to N_2_ through the saturated soil.

## Conclusions

We developed a model to predict the fate, transport and transformation of nitrogen species in a conventional P&S drainfield and in two types of shallow narrow drainfields (PSND and GEO) under current and climate changing conditions. The model was used to gain knowledge about the effect of soil water content on nitrification and denitrification rates in soil drainfields. Based on the calibrated model, UCODE was an efficient tool to determine water flow and transport parameters with acceptable goodness-of-fit between the observed and simulated data. However, when soil water content data are fitted, additional parameter information (i.e., soil hydraulic conductivity, *K*_*s*_) should be gathered to increase the model precision and to provide more realistic results.

The water content dependency functions were suitable to compute nitrification and denitrification rates in all drainfield types. Nevertheless, more research has to be done to determine the relationship between WFPS and relative rate of microbial nitrification and denitrification for STE and ATE. Additionally, this information has to be incorporated in the model to obtain better results.

When the water table is located at shallower distance from the infiltrative surface and under warmer soil temperature conditions, the model predicted that nitrification and denitrification rates are likely to be affected in all drainfield types. Nitrification was restricted (NO_3_^-^ concentrations were reduced) and subsequently N losses as N_2_ were increased between 21% and 37%. The simulation of the fate and transport of N in STAs suggest that climate change may positively influence the performance of OWTS, and also reveals that these treatment systems will response to changing temperature and rising water table conditions, predicted by climate assessment models. These results allow for the quantification the N losses in all OWTS drainfield types and an estimation of the N species fluxes under changing climate conditions. This information is useful to better understand the N transport and transformation mechanisms in OWTS now and in the future.

Our results are more representative for the temperate climates of the U.S. Northeast or Mid-Atlantic region. Both higher and lower initial soil temperatures and corresponding climate changes amplitudes are possible in other parts of the world. Therefore, additional modeling studies, together with field investigations, have to be conducted to determine if other temperature amplitudes than simulated herein would result in similar effects on contaminant removal.

## Supporting Information

S1 Table Select morphological, physical and chemical properties of the soil used in drainfield mesocosms. Values for physical and chemical properties are means (n = 7) ± s.d. Measurements of pH, electrical conductance (EC) and cation exchange capacity (CEC) were made on composite samples. Cooper et al. (2015).
(DOCX)

S2 Table Characteristics of septic tank effluent (STE) and sand filter effluent (SFE) used in our study (n = 26–49). Cooper et al. (2015).
(DOCX)
