# Supplementary material for: Modeling Nitrogen Losses in Conventional and Advanced Soil-Based Onsite Wastewater Treatment Systems under Current and Changing Climate Conditions
Source: PLoS One. 2016 Jun 29;11(6):e0158292. doi: 10.1371/journal.pone.0158292 (PMC4927103; doi:10.1371/journal.pone.0158292)
Supplement: S1 Table — Values for physical and chemical properties are means (n = 7) ± s.d. Measurements of pH, electrical conductance (EC) and cation exchange capacity (CEC) were made on composite samples. Cooper et al. (2015). (DOCX) [file pone.0158292.s001.docx]

|  | | | | | | | | | | | | | |
| --- | --- | --- | --- | --- | --- | --- | --- | --- | --- | --- | --- | --- | --- |
|  | | | | | | | | | | | | | |
| Horizon | Depth | Color | Texture | Particle size distribution | | | Structure | Bulk  density | Porosity | Organic matter | pH | EC | CEC |
|  |  |  |  | Sand | Silt | Clay |  |  |  |  |  |  |  |
|  | cm |  |  | % | % | % |  | g cm^-3^ | % | g kg^-1^ |  | µS | mEq 100 g^-1^ |
| A_p1_  A_p2_ | 0-31 | brown | silt loam | 72±13 | 21±12 | 10.2±0.6 | weak granular to subangular blocky | 1.08±0.06 | 59±2 | 0.5±0.03 | 4.9 | 32.7 | 3.9±0.1 |
| B_w_ | 31-44 | yellowish brown | silt loam | 74±13 | 18±13 | 8.1±1.2 | weak med. subangular blocky | 1.27±0.09 | 52±3 | 0.26±0.05 | 4.9 | 20.6 | 2.4±0.4 |
| 2B_w_ | 44-58 | light olive brown | gravelly loamy sand |  |  |  | weak med. subangular blocky |  |  |  |  |  |  |
| 2C1 | 58-70 | light olive brown | v. gravelly coarse sand; 40% gravel | 96±1 | 1.6±1.1 | 2.8±0.1 | structureless single grain; loose | 1.69±0.08 | 36±3 | 0.05±0.003 | 4.5 | 1.6 | 0.44±0.12 |
| 2C2 | 70-96 | light yellowish brown | v. gravelly coarse sand; 45% gravel | 94±3 | 3.6±3.4 | 2.7±0.1 | structureless single grain; loose | 1.61±0.06 | 39±2 | 0.06±0.02 | 4.4 | 2.7 | 0.48±0.15 |
| 2C3 | 96-130 | pale yellow | coarse sand |  |  |  | structureless single grain; loose |  |  |  |  |  |  |
