# Supplementary material for: Modeling Nitrogen Losses in Conventional and Advanced Soil-Based Onsite Wastewater Treatment Systems under Current and Changing Climate Conditions
Source: PLoS One. 2016 Jun 29;11(6):e0158292. doi: 10.1371/journal.pone.0158292 (PMC4927103; doi:10.1371/journal.pone.0158292)
Supplement: S2 Table — Cooper et al. (2015). (DOCX) [file pone.0158292.s002.docx]

|  | | | | | | | |
| --- | --- | --- | --- | --- | --- | --- | --- |
| Property | STE | | |  | SFE | | |
|  | Median | Range |  | | | Median | Range |
| pH | 6.4 | 5.9 – 7.3 |  | | | 4.7 | 3.2 – 6.1 |
| Dissolved O_2_, mg L^-1^ | 0.0 | 0.0 – 0.4 |  | | | 2.5 | 1.2 – 4.1 |
| BOD_5_, mg L^-1^ | 260 | 120 - 410 |  | | | 19 | 0 – 80 |
| Total suspended solids, mg L^-1^ | 41 | 18 – 89 |  | | | 5.0 | 0.0 – 30 |
| Electrical conductivity, µS | 770 | 550 – 920 |  | | | 560 | 360 – 750 |
| Fecal coliform bacteria, CFU 100 mL^-1^ | 3.6 × 10^5^ | 3.0 × 10^4^ – 4.5 × 10^6^ |  | | | 3.0 × 10^2^ | 6.0 × 10^0^ – 3.9 × 10^4^ |
| *E. coli* CFU 100 mL^-1^ | 3.4 × 10^5^ | 1.0 × 10^4^ – 4.4 × 10^6^ |  | | | 9.2 × 10^1^ | 0 – 3.9 × 10^4^ |
| Total N, mg L^-1^ | 72 | 42 – 95 |  | | | 54 | 29 – 88 |
| NH_4_-N, mg L^-1^ | 56 | 40 – 74 |  | | | 14 | 6.0 – 34 |
| NO_3_-N, mg L^-1^ | 0.1 | 0.0 – 0.9 |  | | | 30 | 10 – 58 |
| Total P, mg L^-1^ | 11 | 6.8 – 17 |  | | | 7.8 | 3.8 – 13 |
| PO_4_-P, mg L^-1^ | 6.4 | 3.3 – 7.9 |  | | | 4.3 | 2.7 – 6.2 |
| SO_4_-S, mg L^-1^ | 0.8 | 0.2 – 7.2 |  | | | 9.3 | 4.2 – 28.8 |
| Collection temperature, °C | 16 | 5 – 22 |  | | | 15 | 4 – 21 |
